# Sedentary Lifestyle Matters as Past Sedentariness, Not Current Sedentariness, Predicts Cognitive Inhibition Performance among College Students: An Exploratory Study

**DOI:** 10.3390/ijerph18147649

**Published:** 2021-07-19

**Authors:** Valentin Magnon, Guillaume T. Vallet, Frédéric Dutheil, Catherine Auxiette

**Affiliations:** 1 Department of Psychology, Université Clermont Auvergne, CNRS UMR 6024, LaPSCo, 63000 Clermont-Ferrand, France; valentin.magnon@uca.fr (V.M.); Guillaume.vallet@uca.fr (G.T.V.); fdutheil@chu-clermontferrand.fr (F.D.); 2 Faculty of Health, School of Exercise Science, Australian Catholic University, Melbourne, VIC 8001, Australia

**Keywords:** sedentariness, sedentary behavior, physical activity, cognition, executive functions

## Abstract

**Background:** Currently, sedentariness is assessed over a short period of time, thus it is difficult to study its cognitive implications. To investigate the cognitive consequences of a sedentary lifestyle, the past level (i.e., the sedentary time accumulated over the years) and current level of sedentariness should be considered. This pilot study aimed to investigate the negative association between a sedentary lifestyle and cognition by considering both the current and past sedentariness. It was expected that the physical activity level moderates the potential negative association between sedentariness and cognition. **Methods:** 52 college students (*M*_age_ = 20.19, *SD*_age_ = 2; 36 women) participated in the study. Current sedentariness (ratio of sedentary time in the last year), past sedentariness (ratio of sedentary time accumulated in previous years), and physical activity (ratio of time spent in physical activity in years) were assessed using a questionnaire. Cognitive inhibition, cognitive flexibility, and working memory updating were measured through three specific tests. **Results:** Past sedentariness significantly explained the inhibition performance when controlled for physical activity, whereas current sedentariness did not. More precisely, past sedentariness only negatively predicted cognitive inhibition when the physical activity level was low (*β* = −3.15, *z*(48) = −2.62, *p* = 0.01). **Conclusions:** The impact of sedentariness on cognitive functioning might only be revealed when past sedentariness and physical activity are controlled.

## 1. Introduction

Compared to their ancestors, modern humans are very sedentary. We are spending increasing amounts of time on activities that require prolonged sitting such as working on a computer, driving, watching television, or even participating in social interactions through the digital social network. Sedentary behavior is defined by “any waking behavior characterized by an energy expenditure ≤1.5 Metabolic Equivalents Tasks, while being in a sitting, reclining or lying posture” [1]. Sedentary behaviors are recognized as a major new health risk with, for example, an association between sitting and type 2 diabetes [2,3], cardiovascular disease [4], musculoskeletal disorders [5], and some cancers [6]. Moreover, sedentary behaviors are positively associated with all-cause mortality, independent of the level of physical activity [7,8], thus supporting a possible causal link between sitting and deleterious health consequences [9,10].

These results also imply that sedentariness and the level of physical activity are two different and independent constructs [11,12,13,14]. Physical activity could be defined as any bodily movement that is produced by the skeletal muscles and results in energy expenditure [15]. According to physical activity recommendations, being physically active requires at least 150 min of moderate-intensity aerobic physical activity throughout the week, at least 75 min of vigorous-intensity aerobic physical activity throughout the week, or an equivalent combination of moderate and vigorous-intensity activity [16]. An individual can then be sedentary even though he/she is engaged in weekly physical activity [12]. Furthermore, despite its health benefits, such as improving glycemic, cardiovascular, and cardiorespiratory functioning, and preventing some cancers [17,18,19], regular physical exercise (i.e., a subset of physical activity that is a planned, structured, and repetitive process that aims to maintain and improve physical fitness, see Reference [15]) seems insufficient to fully protect against the harmful consequences of sedentary behavior [11,20]. Regardless, high levels of physical activity might attenuate the association between sedentary behavior and some negative health consequences (e.g., cardiovascular disease mortality and cancer mortality, see Reference [21]).

Similarly, the same antagonistic effect of physical activity and sedentariness might also be observed at a cognitive level. Indeed, prolonged and regular physical activity is associated with improved cognitive performances regardless of age [22,23,24,25,26]. For instance, adults who have been active for extended periods of time have demonstrated improvements in cognitive flexibility [27], cognitive inhibition [28], and working memory performance [29]. Physical activity would selectively benefit executive functions [28,30,31] such as control processes implemented when the usual courses of action are no longer relevant in a given context (i.e., new, unfamiliar, dangerous, or conflicting situations, see Reference [32]) and allowing the person to adapt to new situations. If physical activity through energy expenditure and muscle contractions [33,34] positively influences cognitive health, a sedentary lifestyle characterized by prolonged sitting and a lack of movement might have the opposite effect on executive functioning. In fact, prolonged sitting appears to be associated with poor executive functioning [35,36].

The negative association between sedentary behavior and cognition is not always reported in the literature and remains controversial [37,38] due to methodological issues such as low statistical power, heterogeneity in the measurements of sedentary behavior and dependent variables [37], heterogeneity of the sample studied, or the absence of controlled variables such as physical activity level [39,40]. However, due to its positive effect on cognition [41,42], physical activity should be controlled when the cognitive implications of sedentary behaviors are studied. Indeed, regular physical activity might at least reduce, if not eliminate, the potential negative relationship between sedentariness and cognition and thus act as a moderator. In other words, because of the cognitive benefits of physical exercise, the strength or direction of the association between sedentariness and cognition would change with the level of physical activity. Furthermore, it was highlighted that the term sedentary behavior is still misused and mistaken for physical inactivity, leading to false conclusions [43,44]. When sedentary behavior is correctly defined and assessed, no effect on cognition is found over a short period of time ([45]), while prospective studies investigating self-reported sedentary time in daily life suggest negative associations with executive functioning (see Reference [46], for opposing results see Reference [47]).

Those differences may be explained by another major limitation of previous studies on the association between sedentariness and cognition: sedentariness was mainly assessed over a short period of time by estimating the time spent sitting in the last few days or so, or at most in the past year [38]. This estimate of current sedentariness is relevant for studying its physiological effects but probably not for studying its cognitive effects as is the case for physical activity [29,48]. Indeed, regular physical exercise should last at least 4 weeks [49] in order to observe any cognitive benefits that could intensify with a longer training duration [50]. Thus, rather than considering the sedentary behaviors produced at a given time, it seems necessary to consider all the sedentary behaviors accumulated over the years (i.e., to consider the level of past sedentariness [38]). Although long-term sedentariness is not yet defined, it seems essential to consider in order to determine its possible psychological and cognitive consequences [51]. For example, the mixed results on the association between sedentary and neurodegenerative diseases might be explained by the lack of a proper definition of sedentariness [44].

In light of these limitations, the present pilot study aimed to investigate the influence of sedentariness as a lifestyle on executive functioning. Indeed, executive functions seem to be selectively influenced by daily life activities [52] such as physical exercise [28,30,31]. A new questionnaire was created both to estimate current sedentary behaviors and to calculate a sedentary lifestyle index (past sedentarity). The questionnaire also allowed for the estimation of the physical activity index that was necessary to consider in the analyses. As sample homogeneity was one of the main factors to consider in determining whether sedentarity has an impact on cognition [38], the study was conducted with college students aged between 18 and 25. College students were chosen not only because they are highly sedentary [53,54,55] but also because they are fairly homogeneous [56] on several levels (e.g., age, use of modern technologies, and cognitive engagement). It was expected that only past sedentariness rather than current sedentariness would be negatively associated with executive functioning, especially when the level of physical activity was low.

## 2. Materials & Methods

### 2.1. Participants

Sixty-three college students (45 women), enrolled in different college courses, and aged between 18 and 25 years old (*M* = 20,33; *SD* = 1.90) were solicited by email and individually participated in this study in exchange for participating in a random draw to win one of four gift certificates worth €100 each. Given the exploratory nature of this study (as past sedentariness has never been studied), no power analysis was conducted. A pre-experimental form was used to collect sociodemographic data (e.g., age, sex, and number of years of education) and medical history (e.g., use of substances or medications). Any factors that may affect cognitive functioning such as neurological (e.g., head trauma or epilepsy), physiological (e.g., hypo or hyperthyroidism or body mass index over 35), or neurodevelopmental (e.g., autism or dyslexia) problems were considered exclusion criteria. Similarly, faster reaction times under more difficult conditions than under easier conditions of the same test were considered as exclusion criteria as they suggest that the participant did not complete the test adequately. Two inclusion tests were also administered: the Verbal Fluency Test [57,58,59] and the French National Adult Reading Test (fNART) [60,61,62], serving as simple indicators of the sociocultural status of participants. Furthermore, the Hospital and Anxiety Depression Scale (HADS) [63,64,65] and the Balanced Inventory of Desirable Responding (BIDR) [66] (French translation, D’Amours-Raymond, 2011) were administered to measure anxiety, depression, and social desirability. These two scales were administered last to reduce the potential threat associated with the questionnaires [67]. A HADS cut-off score of ≥11 was used as a pathological score for the anxiety and depression subscales [64]. Finally, participants were required to be fluent in French and to have normal or corrected-to-normal vision and audition skills.

**Figure 1:**
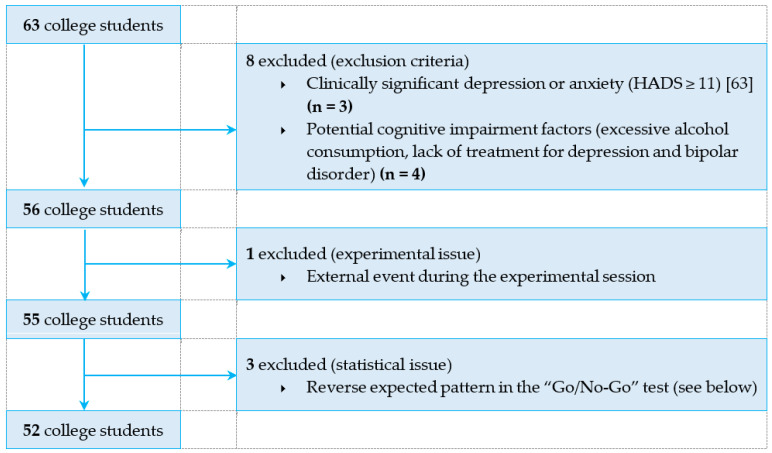
Flow chart of study participants.

Based on these criteria, 11 participants were excluded from the statistical analyses. The reasons for their exclusion are presented in Figure 1. The final sample thus consisted of 52 college students (36 women, see Table 1). The study was approved by the Ethics Committee of the University of Clermont Auvergne (No. IRB00011540-2019-14) and participants signed a consent form guaranteeing anonymity and confidentiality.

### 2.2. Outcome Measures

#### Sedentary and Physical Activity Assessment

A new survey called the Physical Activity and Sedentariness Survey (PASS, Clermont-Ferrand, France) was created to assess the level of physical activity and current and past sedentariness (see Table S1 for the structure of the questionnaire). This questionnaire was developed in accordance with validated sedentary questionnaires [68]. A study not yet published on the PASS has validated the assessment of current sedentariness by comparing the results with the Sitting Questionnaire (SIT-Q) [69], suggesting an adequate validity and reliability of the PASS (*r* = 0.63, *p* < 0.01). It was impossible to assess the validity of the PASS regarding past sedentary time as the present study appears to be the first one to consider past sedentary time in relation to current sedentary time using a questionnaire. Nonetheless, because the estimation of past and current sedentary time is based on the same type of questions, the validity reported for current sedentary time might be extended to past sedentary time. The PASS begins with some open-ended questions on professional occupations and socio-cultural status. Participants were asked to consider their lifetime from the end of high school to compete the PASS as education is mandatory until then in France and because the recall bias might be increased regarding early life memories. The questionnaire is then structured in two main parts. The first part covers the last 365 days (current sedentariness and current physical activity) and the second part covers the previous years (past sedentariness and past physical activity). To simplify the survey structure, current and past physical activity levels are still requested even if one overall physical activity score was used (there is no theoretical reason to distinguish past and current physical activity on their effect on cognition). The questionnaire was designed and operated on Limesurvey [70].

Current sedentariness was defined as the sedentary time over the past year in accordance with validated sedentarity surveys such as the SIT-Q [69]. In addition, this time interval allowed the efficient computation of a ratio reflecting the estimated time spent engaging in sedentary behaviors over the past year. This ratio was assessed by taking into account the last 365 days using questions on the number of weeks of leave, the average time spent sitting on a typical workday, and a typical day off. For instance, participants were asked, “In the last 365 days, during a typical 24 h workday, how many hours did you spend sitting?” Participants were also asked to consider the time spent sitting during meals and transportation.

Current physical activity was assessed by asking questions about the sports participants were engaging in, the average frequency of sports sessions during a working week and a week off, and the average duration of a sports session. For example, participants were asked, “In the last 365 days, have you engaged in at least one sporting activity either in a club or during your leisure time, in a group or individually?”

Past sedentariness was assessed when the participant had a different (more or less sedentary) level of sedentariness compared to their current level. If this was the case, an estimate of the average number of hours spent in a sitting position on a typical day during this period and the length of the period were requested. For example, participants were asked, “How many years did this period, which you spent more (or less) sitting, last?”. Otherwise, when participants indicated the same level of sedentary behavior between the past year and previous life periods, past sedentariness was considered equivalent to current sedentariness.

Finally, with respect to past physical activity, the survey questions focused on periods prior to the current year when participants could have been more or less physically active. If their previous level of physical activity was different from their current level, the average number of hours of exercise in a typical week during this period and the length of the period were requested. For instance, participants were asked, “Do you consider that, in the past, you have experienced a period of more or less intense physical exercise than in the previous year?” Otherwise, the past level of physical activity was considered equivalent to the current one.

For current sedentariness (see Equation (1)), the average number of hours spent in a sitting position on a typical workday was collected for the last 365 days. The same was done for a regular day off. On the basis of these two estimates, the average number of sitting hours per working week and per week of rest were obtained. Subsequently, the result was divided by the total number of waking hours in a year (the time spent sleeping was individually estimated and removed from this base) to obtain the current sedentary ratio.

(1) $$\begin{array}{c} \mathrm{Current}\;\mathrm{sedentariness}\;\mathrm{ratio}=\\ \;\frac{\left[\begin{array}{c} \left({\bar{x}}_{\mathrm{sedentary}\;\mathrm{hours}\;\mathrm{during}\;a\;\mathrm{typical}\;\mathrm{workday}\;}\;*\;\sum^{}\mathrm{workdays}\;\mathrm{over}\;\mathrm{the}\;\mathrm{past}\;\mathrm{year}\;\right)\\ +\left({\bar{x}}_{\mathrm{sedentary}\;\mathrm{hours}\;\mathrm{during}\;a\;\mathrm{usual}\;\mathrm{day}\;\mathrm{off}\;}\;*\;\sum^{}\mathrm{days}\;\mathrm{off}\;\right) \end{array}\right]}{\sum^{}\mathrm{waking}\;\mathrm{hours}\;\mathrm{in}\;a\;\mathrm{year}} \end{array}$$

The past sedentariness (see Equation (2)) was obtained by quantifying the period (number of years) during which a person’s sedentary level differed from their current one. The average number of hours spent in a sitting position on a typical day during this period was then requested. These data allowed for the estimation of the total number of hours spent in a sitting position during this period that was then divided by the total number of waking hours for this period. In France, schooling is mandatory until the age of 16; therefore, the first sixteen years were excluded from the ratio as the level of sedentariness during this period is relatively equivalent from one individual to another. Thus, the estimation of the participants’ past sedentariness began from the high school level.

(2) $$\begin{array}{c} \mathrm{Past}\;\mathrm{sedentariness}\;\mathrm{ratio}=\\ \;\frac{\left[\begin{array}{c} \left({\bar{x}}_{\mathrm{sedentary}\;\mathrm{hours}\;\mathrm{during}\;a\;\mathrm{typical}\;\mathrm{day}\;\mathrm{of}\;\mathrm{of}\;\mathrm{period}\;}\;*\;\sum^{}\mathrm{years}\;\mathrm{that}\;\mathrm{this}\;\mathrm{period}\;\mathrm{lasted}\;\;*\;365\;\mathrm{days}\right)\\ +\left(\sum \mathrm{years}\;\mathrm{sedentary}\;\mathrm{time}\;\mathrm{equivalent}\;\mathrm{to}\;\mathrm{current}\;\mathrm{sedentariness}\;\;*\;\sum \mathrm{current}\;\mathrm{sedentary}\;\mathrm{hours}\right) \end{array}\right]}{\mathrm{Age}\;\;-\;16\;\mathrm{first}\;\mathrm{years}\;} \end{array}$$

Similarly, physical activity was assessed based on the average of both the current (see Equation (3)) and past (see Equation (4)) levels of physical activity. These levels were obtained by calculating the average number of hours of physical activity per week over the last 365 days (current period) and the average number of hours per week over a given period of more or less intense physical activity compared to the current level. As with sedentariness, these hours were divided by the total number of waking hours for each period. As children in France can only start engaging in a sport from the age of 6 and because memory is not well established before the age of 6, the first 6 years were excluded in the calculation. The ratio of past sedentariness and past physical activity were thus calculated on different periods of life as inter-individual differences as the level of physical activity can be observed much earlier (at 6 years old) than for the sedentary level (at the end of high school) as explained above.

(3) $$\begin{array}{c} \;\mathrm{Current}\;\mathrm{physical}\;\mathrm{activity}\;\mathrm{ratio}=\\ \frac{\left[\begin{array}{c} \left({\bar{x}}_{\mathrm{sports}\;\mathrm{sessions}\;\mathrm{during}\;a\;\mathrm{working}\;\mathrm{week}\;}\;*\;\sum^{}\mathrm{working}\;\mathrm{weeks}\;\right)\\ +\left({\bar{x}}_{\mathrm{sports}\;\mathrm{sessions}\;\mathrm{during}\;a\;\mathrm{week}\;\mathrm{off}\;}\;*\;\sum^{}\mathrm{weeks}\;\mathrm{off}\;\right)*\;{\bar{x}}_{\mathrm{hours}\;\mathrm{per}\;\mathrm{session}\;} \end{array}\right]}{\sum^{}\mathrm{waking}\;\mathrm{hours}\;\mathrm{in}\;a\;\mathrm{year}} \end{array}$$

(4) $$\begin{array}{c} \mathrm{Past}\;\mathrm{physical}\;\mathrm{activity}\;\mathrm{ratio}\;=\\ \frac{\left[\begin{array}{c} \left({\bar{x}}_{\mathrm{hours}\;\mathrm{of}\;\mathrm{exercise}\;\mathrm{during}\;a\;\mathrm{typical}\;\mathrm{week}\;\mathrm{of}\;\mathrm{of}\;\mathrm{period}\;}\;*\;\sum^{}\mathrm{years}\;\mathrm{that}\;\mathrm{this}\;\mathrm{period}\;\mathrm{lasted}\;\;*\;52\;\mathrm{weeks}\right)\\ +\left(\sum \mathrm{years}\;\mathrm{during}\;\mathrm{which}\;\mathrm{the}\;\mathrm{physical}\;\mathrm{current}\;\mathrm{level}\;\mathrm{was}\;\mathrm{equivalent}\;\mathrm{to}\;\mathrm{the}\;\mathrm{current}\;\mathrm{one}\;\;*\;\sum \mathrm{current}\;\mathrm{hours}\;\mathrm{of}\;\mathrm{physical}\;\mathrm{activitity}\right) \end{array}\right]}{\mathrm{Age}\;\;-\;16\;\mathrm{first}\;\mathrm{years}\;} \end{array}$$

### 2.3. Cognitive Assessment

Cognition was assessed by using three neuropsychological tests, each measuring one executive function.

#### 2.3.1. Cognitive Flexibility 

The ability to switch from one mental operation to another was assessed by the “Plus-Minus” test [71] that consists of 3 lists of 30 digits. For the first list, participants were asked to add 3 to each digit. For the second, they were asked to subtract 3 from each digit. Finally, for the third list they were asked to alternate between addition and subtraction. Each list should be completed as quickly and accurately as possible. Flexibility was estimated by the difference between the time required to complete the alternation list and the average time required to complete the addition and subtraction lists.

#### 2.3.2. Working Memory Updating 

To assess the ability to update the information in the working memory, participants performed the “2 n-back” task [72] in which numbers were presented in rapid succession; they were asked to indicate whether the current number corresponded to the second to last number presented. This task was executed on the OpenSesame software (Amsterdam, The Netherlands, see [73]). Each number was displayed on the screen for 1500 ms. The working memory updating was estimated by the number of correct answers.

#### 2.3.3. Cognitive Inhibition 

Finally, the capacity to inhibit a predominant response was measured with the “Go/No-Go” task [74]. This computerized task was performed on the OpenSesame software [73] and participants saw individual letters presented successively for 1000 ms each on a computer screen. Under the “Go” condition, participants were asked to press the space bar as quickly as possible as soon as a letter appeared on the screen regardless of the letter. In the “No-Go” condition, they were asked to press the space bar as quickly as possible when a letter was presented on the screen (“Go” stimuli) except when the letter was an “X” (“No-Go” stimulus). Four lists of 20 letters (two for the “Go” condition with 10 times the “X” and two for the “No-Go” condition) were displayed randomly on the screen between participants. The letters were also randomized in the lists. The main result of the “Go/No-Go” task was the commission error rate (making a “Go” response on “No-Go” trials); fewer errors meant better inhibition of the response. Reaction times or the number of correct response in go-trials and omission errors (i.e., falsely not pressing the button in go-trials; also called misses) can also be calculated but they are usually not considered as indices of inhibitory control [75].

### 2.4. Procedure

In individual sessions, an anamnestic interview between the participant and the experimenter allowed the sociodemographic data (age and sex) and medical history of interest to be collected. The participant was then asked to complete the questionnaire on both current and past sedentariness and physical activity. Then, the “n-back”, “verbal fluency”, “Plus-Minus”, “fNART”, and “Go/No-Go” tasks were administered in this order. Finally, participants were asked to complete the BIDR and HADS questionnaires. The researcher stayed with the participant during the whole session. Sessions lasted approximately 50 min.

### 2.5. Statistical Analyses

All statistical analyses were performed using the R software (Rstudio Team, Boston, MA, USA, see [76]). Three independent and continuous variables were obtained from the survey on physical activity and sedentariness: current sedentariness ratio, past sedentariness ratio, and average physical activity ratio. In order to make these scores comparable, they were transformed into z-scores. As the correlation analysis did not reveal any strong relationship between the different executive scores, each was used independently. Social desirability as assessed by the BIDR would have been used as a covariate if it was correlated with any of the variables of interest.

Stepwise multiple linear regression analyses were performed for each executive score with both current and past levels of sedentariness as predictors by entering each variable independently. This was conducted in order to assess which of these factors may account for executive performance. The assumptions for this analysis were tested by using variance inflation factor (VIF) values to check the absence of multicollinearity. The Durbin Watson (DW) value for the independence of errors, the skewness, and kurtosis values for normality were used. The assumptions of variance different from 0 and homoscedasticity were also checked. Only the “Go/No-Go” and “Plus-Minus” tests were used in the analyses. With a kurtosis of 3.58 (*SD* = 0.65) and a skewness of −1.95 (*SD* = 0.33), the “N-Back” scores did not meet the normality assumptions despite a logarithm, square, or reverse transformation of the data. Thus, only the “Go/No-Go” and “Plus-Minus” tests were used in the analyses. Finally, to test whether physical activity moderates the potential association between past sedentariness and executive functioning, a moderation model was used on cognitive inhibition and cognitive flexibility. The package used was “Medmod” [77]. In this package, the moderation analysis was conducted through a multiple regression analysis with an interaction term between a predictor and a moderator in which the moderating effect on the dependent variable was tested 1 SD above and 1 SD below the mean. The predictor was past sedentariness and the moderator was the average level of physical activity. Another equivalent analysis was conducted with current sedentariness as a predictor. The bootstrapping method [78] was used for all moderation analyses.

All data are available at the Center for Open Science (Magnon, Vallet, Dutheil, and Auxiette, Clermont-Ferrand, France, 2020).

## 3. Results

### 3.1. Regression Analyses

There was no outlier defined by a value of ±3 z-scores [79]. Moreover, a diagnosis for leverage and influential data points revealed acceptable data validity. The assumptions were checked in order to perform a multilinear regression analysis. The assumption of variance different from 0 (“Plus-Minus” z-scores, *s*^2^ = 1.02; “Go/No-Go” z-scores, *s*^2^ = 0.93; current sedentariness ratio, *s*^2^ = 0.03; and past sedentariness ratio, *s*^2^ = 0.02), normality distribution with kurtosis, and skewness between −2 and +2 [80], homoscedasticity were met for cognitive inhibition, and cognitive flexibility performances. Furthermore, there was no multicollinearity (current sedentariness ratio, tolerance = 0.72, *VIF* = 1.40 and past sedentariness ratio, tolerance = 0.72, *VIF* = 1.40). Correlation analyses revealed that social desirability was not related to any of the variables of interest, thus the BIDR scores were not used as a covariate. Other factors such as depression or anxiety levels and age were controlled using inclusion criteria. Finally, gender had no effects on any of the variables of interest (i.e., cognitive inhibition and flexibility, past and current sedentariness, and average level of physical activity).

#### Cognitive Flexibility and Cognitive Inhibition

The stepwise multilinear regression analysis (see Table 2) revealed that current and past sedentariness did not explain a significant amount of the variance of z-scores either in the “Plus-Minus” cognitive flexibility test (*F*(2, 49) = 0.44, *p* > 0.10, *R*^2^ = 0.02) or in the “Go/No-Go” cognitive inhibition test (*F*(2, 49) = 0.74, *p* > 0.10, *R*^2^ = 0.01). The data also respected the assumption of independent errors (*DW* = 2.17, *p* > 0.10 for the “Plus-Minus” test, and *DW* = 1.44, *p* > 0.05 for the “Go/No-Go” test).

### 3.2. Moderation Analyses

#### 3.2.1. Cognitive Flexibility 

The moderation analysis conducted for cognitive flexibility revealed that the interaction term between past sedentariness and average physical activity did not account for a significant proportion of the variance in the z-scores of the “Plus-Minus” test (*β* = −25.69, *z*(48) = −0.69, *p* > 0.10, *CI*: −115.64; 37.33). The same moderation model was conducted with current sedentariness as a predictor. The interaction term between current sedentariness and average physical activity did not explain a significant proportion of the variance in the z-scores of the “Plus-Minus” test (*β* = −33.31, *z*(48) = −1.04, *p* > 0.10, *CI*: −128.57; 0.99).

#### 3.2.2. Cognitive Inhibition

**Figure 2:**
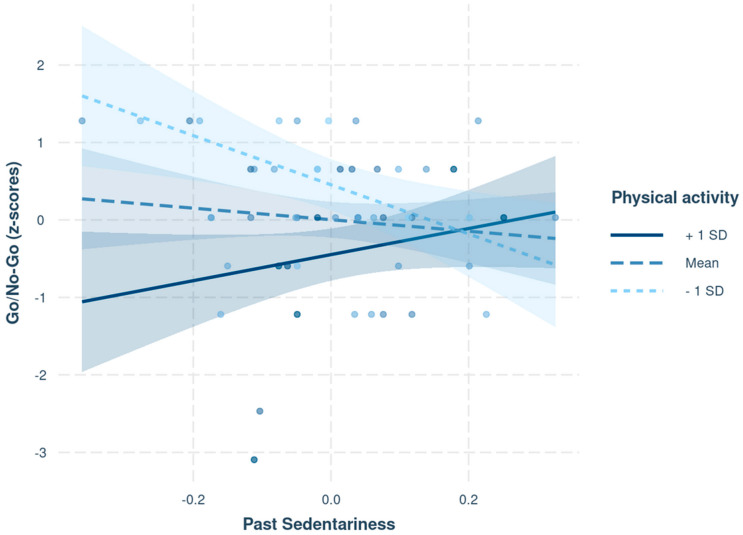
Prediction of z-scores for cognitive inhibition (“Go/No-Go” test) according to past sedentariness for different levels of physical activity (average, low, and high).

The statistical model with past sedentariness as a predictor and physical activity as a moderator was statistically significant *F*(3, 48) = 7.16, *p* < 0.01, *R*^2^ = 0.27. The moderation analysis conducted for cognitive inhibition demonstrated that a significant amount of the variance in z-scores obtained for the “Go/No-Go” test was explained by the interaction term (see Figure 2) between past sedentariness and average physical activity (*β* = 60.49, z(48) = 2.12 *p* = 0.03, *CI:* 12.99; 126.72). Past sedentariness only negatively predicted z-scores in the “Go/No-Go” test when the physical activity level was low (*β* = −3.15, *z*(48) = −2.62, *p* = 0.01, *CI*: −5.42; −0.83). Another moderation model was performed with current sedentariness as a predictor. The interaction term between current sedentariness and average physical activity did not explain a significant proportion of the variance in the z-scores of the “Go/No-Go” test (*β* = 39.28, z(48) = 1.52, *p* > 0.10, *CI:* −2.27; 111.94).

## 4. Discussion

This study aimed to investigate the possible link between sedentariness and cognition by comparing current sedentariness with past sedentariness while controlling for physical activity. In accordance with previous studies [81,82], the regression analyses did not reveal any effects of past or current sedentariness on executive performance when the physical activity level was not consider. However, as hypothesized, when the physical activity level was included in the statistical model, past sedentariness and physical activity explained a statistically significant proportion of the variance for cognitive inhibition performance (but not for cognitive flexibility performance, while working memory updating was not tested due to assumption violation). Thus, participants with low levels of physical activity exhibited poor cognitive inhibition performances when their past sedentariness was high.

To our knowledge, this study is the first to consider the level of past sedentariness in addition to current sedentariness. So far, sedentariness has mainly been assessed over a very short period of time [38] in accordance with the consensual definition of sedentary behaviors [1]. However, our results support the fact that current sedentariness is not sufficient to study the cognitive implications of a sedentary lifestyle as only past sedentariness significantly predicts some of the inhibition performance. This highlights the relevance of defining sedentariness as a lifestyle to investigate its cognitive implications. The second strength of this study is that physical activity was considered a potential moderator, which was not the case in previous research (even if it was controlled in some studies, see for instance Reference [83]). When the participants’ level of physical activity was high, past sedentariness no longer allowed negative predictions of inhibition performance. This supports the hypothesis that physical activity might have a protective effect against the cognitive effects of sedentariness. Increased brain neurotrophic factors (e.g., the brain-derived neurotrophic factor) may be the physiological mechanism underlying the protective effect of physical activity on cognition [84]. Interestingly, exercise intensity and duration appear to influence neurotrophic factors levels, suggesting that relatively high intensity and long-duration exercise may be particularly effective in protecting cognitive functioning [85]. Finally, with respect to the heterogeneity of the sample identified in previous studies [37,38], this research focused on college students as they are fairly homogeneous on several levels (e.g., use of modern technologies and cognitive engagement) while also considering social desirability, anxiety and depression, medical history, and age. The fact that past sedentariness negatively predicts cognitive inhibition when physical activity levels are low in these young adults is therefore relevant. It is worth noting that an important proportion of sedentary behaviors occurring at universities should be considered cognitive engagement behaviors (e.g., learning, reading, and studying) and thus these factors are likely to promote cognitive functioning [36,86]. This may be why the association between sedentary behavior and academic performance has not been established in other studies conducted with college students [81,82].

Concerning the limitations of this study, the data obtained for working memory updating reached the maximum (ceiling effect) and the scores were then not normally distributed. In this study, we used the “2-back” test because the “1-back” test was considered too easy and the “3-back” test too hard. Thus, it seems difficult to find the appropriate test that may be used across different populations. Another common paradigm used to test working memory includes complex span tasks [87,88]. However, according to Miyake’s model [89], the “n-back” task is a better indicator of working memory updating than complex span tasks that reflect the capacity of working memory (the number of items that can be stored and manipulated actively into memory [90]). Another limitation is the way in which sedentariness was assessed. A new questionnaire was created (PASS) that has not been validated and whose psychometric properties have not yet been published. The use of an accelerometer rather than a questionnaire [35,37] is typically recommended because in the latter case respondents are often led to underestimate their time spent sitting [91] and to overestimate their physical activity [92]. However, this solution appears almost impossible to implement in our pilot study as current sedentarity was assessed over the past 12 months and past sedentarity over all previous life periods. Therefore, participants should wear an accelerometer throughout their whole life to obtain objective and real data on their sedentary habits, notably not an appropriate option. However, this limitation is not a real issue as the study focused on the contrast between current and past sedentariness. For each participant current sedentariness was contrasted with past sedentariness individually (within subjects) and participants were of similar age. Thus, any recall bias should be relatively similar for a given participant and should not explain the results observed. Finally, the sample size in this pilot study is relatively small, underscoring the need for further study.

Taken together, our findings suggest that future studies on the relationship between sedentariness and cognition should not only assess current sedentariness but also past sedentariness. They should also consider the level of physical activity as a potential confounding factor that must be controlled for. Beyond the academic setting, the focus can also be placed on the workplace because the work environment is an appropriate situation for intervention protocols [93] in which the reduction of sedentary periods can be beneficial to cognitive functioning [94,95]. Indeed, moving from one’s desk regularly to break sedentary behavior or working while standing is more conceivable in the workplace than taking a 15 min break for physical exercise. However, methodological issues (e.g., duration of the intervention, heterogeneity of the sample, lack of physical activity control, and heterogeneity of cognitive and sedentary time assessment) may limit the understanding of the cognitive implications of sedentary time at work if they are not controlled for [38]. In addition, workers are of all ages and usually older than college students. Thus, they should have accumulated more sedentary time over the years that should increase the potential effect of sedentariness on cognition. For this reason, another population of interest is the elderly who likely possess a greater amount of years of sedentariness. The potential negative effects of sedentariness on cognition could then be particularly noticeable in this population. In support of this hypothesis, a negative relationship between sedentariness and executive functioning has been reported among seniors [35,96] beyond the normal effect of age.

Finally, future studies should examine the criteria for distinguishing sedentary from non-sedentary persons in order to establish sedentariness guidelines. Indeed, future research on sedentariness should help in defining it as a way of life by establishing the number of hours of sedentariness that are detrimental to cognitive health and the amount of physical exercise that can prevent it [51]. Such guidelines could have a positive impact on primary and secondary prevention interventions aimed at reducing the risk of mortality associated with sedentariness [97] by targeting not only the physiological but also the cognitive implications of such a lifestyle (e.g., a possible increased risk of dementia, see References [38,98]). We believe it is important for future research to determine health criteria regarding sedentary time in relation to other recognized risk factors to better understand the long-term impact of sedentariness on physical and cognitive health [99]. In addition to promoting the importance of regular physical exercise, sitting guidelines would improve the efficacy of interventions such as the use of active workstations that do not seem to decrease work-related performance [100] or break up prolonged sitting times by standing for a few minutes [101]. This type of intervention would not only be relevant in the workplace, but may also be generalized to universities and schools during class.

The results of this study highlight the need to consider physical activity as a protective factor and a means of reducing sedentary time, as well as the need to define sedentariness as a way of life in order to study its cognitive consequences. However, there is currently no definition of a sedentary lifestyle. We believe it is important that future research focuses on establishing criteria that distinguish sedentary from non-sedentary people in order to define such a lifestyle and prevent possible related health issues. One population of interest that has not yet been the subject of in-depth studies on the consequences of sedentary time on cognition is that of workers. If sedentariness at work is indeed harmful to cognitive health, it will become urgent to propose interventions to reduce it.

## 5. Conclusions

In this study, we examined the relationship between sedentariness and cognition under the possible influence of physical activity level on this relationship. The main asset of this research was the creation of a new survey to measure current and past sedentariness, a construct that has not previously been considered. Another strength was the use of the level of physical activity as a moderator. Past sedentariness only negatively predicted cognitive inhibition performances when the physical activity level was low. Thus, two possible conclusions can be drawn. Firstly, current sedentariness is not sufficient to reveal the negative impact of sedentariness on cognition and past sedentariness must also be considered. Secondly, physical activity might have a protective effect against the deleterious cognitive consequences of sedentariness. One limitation of our study is that the current sedentariness could have been measured using a more objective means than a survey (e.g., past sedentrainess however could not be assessed by an accelerometer). Moreover, as past sedentariness is quite difficult for participants to estimate, a recall bias may exist. The consideration of past sedentariness may change how it should be defined and assessed. Indeed, consensual criteria to define sedentariness as a lifestyle are still missing. A better understanding of sedentariness could benefit preventive interventions and reduce the mortality risk associated with the health issue that is sedentariness.

## Figures and Tables

**Table 1:** Description of the demographic characteristics (mean and standard deviation in brackets), current and past sedentariness ratios, and average physical activity ratio of the final sample.

Variables	Mean (SD)
N (F/M)	52 (36/16)
Age	20.29 (2.00)
Education	14.80 (1.81)
fNART	22.40 (3.94)
Verbal fluency	20.11 (3.60)
BIDR	62.40 (6.58)
BMI	22.25 (3.63)
HADS anxiety level	7.65 (3.08)
HADS depression level	3.46 (2.11)
Current sedentariness ratio ^a^	0.48 (0.16)
Past sedentariness ratio ^b^	0.55 (0.14)
Mean physical activity ratio ^c^	0.04 (0.04)

Abbreviations: F = Female; M = Male; fNART = French National Adult Reading Test; BIDR = Balanced Inventory of Desirable Responding; BMI = Body Mass Index; and HADS = Hospital Anxiety/Depression Scale. ^a^ Ratio of time spent in a sitting position in the last 365 days. ^b^ Ratio of time spent in a sitting position in years prior to the current year, excluding the first 16 years (see Section 2.2 for further details). ^c^ Ratio of time spent in physical activity from age 6 to the day of the study (see Section 2.2 for further details).

**Table 2:** Multiple linear regression coefficients on z-scores of the “Go/No-Go” (cognitive inhibition) and “Plus-Minus” (cognitive flexibility) tests for the current and past sedentariness ratio.

	Predictors	*β*	*t*	*p*
Plus-Minus Test *^a^*	Current sedentariness	−0.29	−0.28	0.78
	Past sedentariness	−0.74	−0.61	0.54
Go/No-Go Test *^b^*	Current sedentariness	0.90	0.91	0.37
	Past sedentariness	−1.35	−1.17	0.25

*^a^* Constant: *β* = 0.55; *t* = 0.92; *p* = 0.36; *^b^* Constant: *β* = 0.34; and *t* = 0.60; *p* = 0.55.

## Data Availability

All data are available at the Center for Open Science (https://osf.io/rcgep/, accessed on 3 April 2019).

## Supplementary Materials

The following are available online at https://www.mdpi.com/article/10.3390/ijerph18147649/s1.
