# Supplementary material for: Sedentary Lifestyle Matters as Past Sedentariness, Not Current Sedentariness, Predicts Cognitive Inhibition Performance among College Students: An Exploratory Study
Source: Int J Environ Res Public Health. 2021 Jul 19;18(14):7649. doi: 10.3390/ijerph18147649 (PMC8303919; doi:10.3390/ijerph18147649)
Supplement: Supplementary file 1 [file ijerph-18-07649-s001.zip › ijerph-1285305-supplementary.pdf]

International Journal of
Environmental Research and Public Health

MDPI

Article

Sedentary Lifestyle Matters as Past Sedentariness, Not Current Sedentariness, Predicts Cognitive Inhibition Performance among College Students: An Exploratory Study

Valentin Magnon [1], Guillaume T. Vallet [1], Frédéric Dutheil [1,2] and Catherine Auxiette [1,*]

[1] Department of Psychology, Université Clermont Auvergne, CNRS UMR 6024, LaPSCo, 63000 Clermont-Ferrand, France; valentin.magnon@uca.fr (V.M.); Guillaume.vallet@uca.fr (G.T.V.); fdutheil@chu-clermont-ferrand.fr (F.D.)

[2] Faculty of Health, School of Exercise Science, Australian Catholic University, Melbourne, VIC 8001, Australia

* Correspondence: catherine.auxiette@uca.fr

Citation: Magnon, V.; Vallet, G.T.; Dutheil, F.; Auxiette, C. Sedentary Lifestyle Matters as Past Sedentariness, Not Current Sedentariness, Predicts Cognitive Inhibition Performance among College Students: An Exploratory Study. *Int. J. Environ. Res. Public Health* **2021**, *18*, 7649. https://doi.org/10.3390/ijerph18147649

Academic Editor: Paul B. Tchounwou

Received: 18 June 2021
Accepted: 15 July 2021
Published: 19 July 2021

Publisher's Note: MDPI stays neutral with regard to jurisdictional claims in published maps and institutional affiliations.

Copyright: © 2021 by the authors. Submitted for possible open access publication under the terms and conditions of the Creative Commons Attribution (CC BY) license (http://creativecommons.org/licenses/by/4.0/).

Table S1. Structure of the Physical Activity and Sedentariness Survey (PASS).

**Introduction**
**1.** **Status.** The participant was asked whether or not they were a student and whether they were working in at least one profession (being a student is considered a professional activity). e.g., "This past year, did you engage in one or more occupational activity?"
**2.** **Studies and professional occupation(s).** Taking into account the last 365 days, the participant was asked to indicate 1) the nature of their college studies (if any); 2) if applicable, the number of professional occupations they were engaged in; 3) the number of half-days per week for each professional activity and/or study; 4) the type of professional occupation(s); 5) the length of time (in months and years) they had been engaged in each professional occupation and/or study. e.g., "In a typical working week, how many half-days did you engage in this (or these) occupational activity(ies)?"
**Current time period (the last 365 days)**
**3.** **Current sedentariness.** In this section, the number of weeks off in the past year, the mean sedentary hours per typical 24 h working day and per typical day off (if different) were requested. e.g., "In the last 365 days, during a typical 24 h working day, how many hours did you spend sitting?"
**4.** **Sedentary activities.** The participant was asked how many hours they spent in several sitting activities (e.g. working on a computer, watching TV, reading, playing video games, etc.) during a typical working day and during a typical day off (if different). This section was not used in our analysis. e.g., "This past year, during a typical 24 h working day, how long did you sit while doing the following activities?"
**5.** **Current physical activity.** In this section, the participant was asked if they engaged in any physical exercise, the number and type of exercise, the number of sessions and the average duration of a typical exercise session. e.g., "In the last 365 days, have you taken part in at least one sporting activity either in a club or during your leisure time, in a group or individually?"
**Past time period (prior to the last year)**
**6.** **Past sedentariness.** The participant was asked if, in previous years, their level of sedentariness differed from that of the last year. If so, they were asked for the average number of sedentary hours in a typical day during this period and the number of years it lasted. The participant was informed that they should only consider the period after the end of high school (16 years old). The type of sedentary activities was also requested, but the results of these data have not been presented here. e.g., "The period where you spent more (or less) time sitting lasted for how many years?"
**7.** **Past physical activity.** The participant was asked if they had experienced a period of more or less exercise than the previous year. If so, the duration in years and the average number of hours spent exercising in a typical week were collected. The type of physical exercises was also requested, but the results of these data have not been presented here. e.g., "In the past, do you consider that you have experienced a period of more or less intense physical exercise than in the past year?"
**Supplementary information**
**8.** **Educational background.** The highest diploma or degree, whether the participant did an apprenticeship or traineeship and the kind of apprenticeship (if applicable) were collected. e.g., "Did you do an apprenticeship or a traineeship during your high school years?"
**9.** **Sleep quantity.** The participant was asked to indicate the average number of hours of sleep per night. "Since you were 18, how many hours do you sleep on average per night?"
**10.** **Age.** The participant was asked to indicate their age. "How old are you?"
